# Carbapenem resistance in *Enterobacterales* from agricultural, environmental and clinical origins: South Africa in a global context

**DOI:** 10.3934/microbiol.2023034

**Published:** 2023-09-25

**Authors:** Taish Ramkisson, Diane Rip

**Affiliations:** Department of Food Science, Stellenbosch University, Stellenbosch, 7600, South Africa

**Keywords:** carbapenemase-producing *Enterobacterales*, carbapenem resistance, β-lactamases, Africa, agricultural, food-animals, clinical

## Abstract

Carbapenem agents are regarded as last-resort antibiotics, however, bacterial resistance towards carbapenems has been reported in both clinical and agricultural settings worldwide. Carbapenem resistance, defined as the resistance of a bacteria towards one or more carbapenem drugs, can be mediated in either of, or a combination of, three mechanisms–although, the mechanism mediated through the production of carbapenemases (β-lactamases that are able to enzymatically degrade carbapenems) is of most significance. Of particular concern is the occurrence of carbapenemase producing *Enterobacterales* (CPE), with literature describing a dramatic increase in resistance globally. In South Africa, increases of carbapenemase activity occurring in *Enterobacter* species, *Klebsiella pneumoniae*, *Acinetobacter baumannii* and *Pseudomonas aeruginosa* have recently been reported. CPE can also be found in agricultural environments, as global studies have documented numerous instances of CPE presence in various animals such as pigs, cattle, seafood, horses and dogs. However, most reports of CPE occurrence in agricultural settings come from Northern America, Europe and some parts of Asia, where more extensive research has been conducted to understand the CPE phenomenon. In comparison to clinical data, there are limited studies investigating the spread of CPE in agricultural settings in Africa, highlighting the importance of monitoring CPE in livestock environments and the food chain. Further research is necessary to uncover the true extent of CPE dissemination in South Africa. This review will discuss the phenomenon of bacterial antibiotic resistance (ABR), the applications of the carbapenem drug and the occurrence of carbapenem resistance globally.

## 1. Introduction

The rapid increase and emergence of bacterial antibiotic resistance (ABR) poses a great threat to human health worldwide, being one of the foremost challenges of our century. Environmental hotspots of bacterial ABR include several aquatic environments and terrestrial environments [1],[2] . Some food and drinking water supplies have also been identified as bacterial ABR hotspots resulting in human infections [3],[4]. The presence and dissemination of bacterial ABR in food-production settings also poses a great risk to public and private healthcare and therefore need to be evaluated against the clinical settings to mitigate ABR-associated illnesses and deaths [5]–[7]. ABR in foodborne pathogens is becoming increasingly more detrimental as first-line antibiotics may no longer be capable of treating food-borne illnesses, limiting treatment options [5],[8].

Carbapenems are a class of β-lactam antimicrobial agents, which function by interfering with the synthesis of bacterial cell walls by binding and inactivating penicillin-binding proteins (PBPs) [9]–[11]. Carbapenem antimicrobials are vital for human health as they are the last line of defence drugs against multi-drug resistant bacteria [12],[13]. Selective pressure is the effect applied by some factor, such as antimicrobials, on the natural selection of an organism and thus promoting one group of organisms over another. Beta-lactam antimicrobials (other than carbapenems) are used in veterinary medicine owing to their high specificity and selective toxicity, leading to the overuse of these antibiotics and thus creating the selective pressure for carbapenem (prohibited in livestock production) resistance [14],[15]. One mechanism of bacterial resistance towards carbapenem antimicrobials is the production of carbapenemases by bacteria [16]. Carbapenemases, a class of β-lactamases, are enzymes that can destroy carbapenem antibiotics allowing bacteria to resist antibiotic treatment. These enzymes can be found in strains of *Enterobacterales*, which are commonly referred to as carbapenemase-producing *Enterobacterales* (CPE). The *Enterobacterales* order consists of gram-negative bacteria such as *Salmonella, Escherichia, Klebsiella, Enterobacter, Serratia, Proteus, Shigella* and more [17]–[19]. Gram-negative bacteria particularly are harder to destroy due to the presence of their double cell membranes and multidrug resistance advantages [11],[20]. CPE can occur in agricultural settings with research reporting many cases of CPE presence in pigs, cattle, seafood, horses, dogs and so forth from across the globe [18],[21]–[24]. Although most people are not exposed directly to livestock, enteric microflora from livestock animals can contaminate fresh and raw meat products that are then distributed to consumers and retail markets over broad regions [21],[25]. Therefore, CPE presence in livestock animals can potentially result in the infection of consumers or foodborne outbreaks.

The prevalence of carbapenem resistance by *Enterobacterales* is increasing worldwide as well as within South Africa (SA). The *Antibiotic Resistance Threats in the United States* report by the Centers for Disease Control and Prevention (CDC) in 2019, projected that CPE prevalence will be a great health concern in the world in the future. CPE prevalence was categorised as an urgent threat by the report, which is the most concerning level [26]. While the dissemination of CPE in Europe, North America and Asia has been well documented, the true extent of CPE prevalence in Africa has not yet been fully explored. Due to the lack of antimicrobial resistance surveillance systems in Africa, comprehending the extent of CPE is unlikely. However, there are already some clinical reports of CPE occurring throughout Africa within *E. coli, Enterobacter* species, *S. marcescens, K. pneumoniae* and *Citrobacter* species [18],[24],[27]–[29]. Studies regarding CPE dissemination within agricultural settings in Africa and SA are very few, which indicates the dire need for CPE surveillance in African livestock settings as well as food chains [27].

The National Institute for Communicable Diseases (NICD) in SA, which monitors carbapenem-resistant *Enterobacterales* (CRE) in clinical settings, does not survey CRE from environmental or livestock sources. However, yearly NICD communiqués have identified the occurrence of CRE in clinical settings in both private and public sectors within SA [22].

## 2. Antibiotic resistance and surveillance in Africa

An estimated 700 000 deaths occur annually from ABR bacteria-related illnesses, and this number is said to reach 10 million by the year 2050 if no action against bacterial ABR is taken [16]. Antibiotic resistance places a burden on individuals and on public health settings as there is an increase in associated deaths, increased infections and severity of infections, higher rates of failed treatments and higher costs of hospital services [30],[31].

Information obtained from bacterial ABR surveillance systems allows for the study of antimicrobial susceptibility data, which can be used to control and prevent ABR as well as guide empiric treatment for hospital-related infections in healthcare facilities. The first global report on bacterial ABR surveillance in 2014 by the World Health Organisation (WHO) emphasised a substantial lack of bacterial ABR surveillance information and a paucity of standards for surveillance methodology worldwide [7],[32]. This report demonstrated an increasing phenomenon of bacterial resistance in the African continent, with a significant number of transmissible antibiotic-resistant bacteria in hospitals and communities [33]. African reviews suggest that there are trends of resistance towards commonly used antibiotics by various pathogens such as *K. pneumoniae*, *E. coli*, *Shigella* species, *Vibrio cholerae* and *Salmonella* species [16],[18],[29],[34]. With the insufficient information regarding the bacterial ABR crisis in Africa [7],[35] , it is vital that surveillance be properly established to reduce ABR prevalence in a region with high numbers of immunocompromised individuals who suffer from malnutrition, poor sanitation and human immunodeficiency virus/acquired immunodeficiency syndrome [30].

A systematic review of twelve published articles on bacterial ABR in eastern Africa from 2005 to 2015 identified resistance to commonly used antibiotics and the rapid growth of multidrug-resistant bacteria in the region [2]. The authors further identified a severe dearth of information regarding bacterial ABR as well as hospital-acquired infections, suggesting that rigorous investigation and surveillance are necessary. Similar findings were reported in another systematic review, where the authors studied antibiotic-resistant *Salmonella* and *E. coli* from 2004 to 2015 in Tanzania. They observed an increase in ABR and limited reliable data detailing the degree of resistance. The authors recommended that antibiotic stewardship and infection control measures are vital and should be applied using an established and standardised surveillance system [36]. Antibiotic stewardship programmes are important measures that ensure judicious use and prescribing practices for antibiotics in healthcare sectors, thereby reducing the prevalence of antibiotic-resistant infections and colonisation [12],[37].

Authors of a study surveyed the antimicrobial usage in animals in SA, focusing on food animals [38]. They identified authorised in-feed antimicrobials allowed for use as growth promoters for animals in SA. The antimicrobial classes included tetracyclines, macrolides and lincosamides and pleuromutilins, quinoxalines, polypeptides, nitrofurans, ionophores, streptogramins, glycolipids, oligosaccharides, phosphonic acids and polymeric compounds. Carbapenem antibiotics are not listed and are not authorised for use as in-feed growth promoters in SA, which corresponds with the list of authorised antimicrobials of veterinary importance around the globe described by the *World Organisation for Animal Health list of antimicrobials of veterinary importance* [39]. Given that carbapenem use in food-producing animals is not licensed, residues of carbapenem antimicrobials in foods of animal origin should be absent [19]. Knowledge and data on antibiotic sales used for husbandry are scarce in SA, hindering the identification of trends and patterns related to ABR [31].

In response to an increase in bacterial ABR-associated illnesses and deaths, several organisations have been established to reduce the bacterial ABR crisis in SA. The South African Antibiotic Stewardship Programme is one of these organisations, which consists of various healthcare experts who aim to ensure the judicious administration of antibiotics and to educate others on bacterial ABR [40]. Additionally, the Department of Health (DoH) implemented a bacterial ABR strategy framework plan from 2014 to 2019. The objectives of the strategy improved bacterial ABR surveillance and documentation, and implemented antimicrobial stewardship programmes [40].

Although SA is the second largest economy in Africa with many more resources when compared to other African countries, the quality of bacterial ABR surveillance systems differs greatly amongst institutions across the country, with surveillance system standards not being implemented in most South African healthcare settings [41]. Effective surveillance programmes could aid policymakers in developing national treatment guidelines, prevention and control protocols and antibiotic stewardship programmes.

## 3. Carbapenem resistance and clinical impact

Carbapenem resistance is the resistance of a bacteria towards one or more carbapenem drugs [42]. Many gram-positive bacteria (such as *Streptococcus* species and *Staphylococcus* species), non-fermenting gram-negative bacteria (such as *Acinetobacter* species and *Pseudomonas* species) and *Enterobacterales* (such as *E. coli* and *K. pneumoniae*) have become increasingly resistant to carbapenem antimicrobials [29],[43]–[46]. Microbial resistance to carbapenems either occurs when membrane permeability variations arise due to the loss of specific outer membranes or to the gain of efflux pumps, or when bacteria are able to develop or acquire structural changes within their PBPs, or when bacteria obtain metallo-beta-lactamases (MBLs), which can enzymatically degrade carbapenems [11],[47],[48]. A combination of these resistant mechanisms can result in elevated levels of carbapenem resistance, as seen in specific bacterial species such as *A. baumannii*, *P. aeruginosa* and *K. pneumoniae* [47],[49].

Carbapenem resistance in gram-positive cocci distinctly varies from gram-negative rods, as carbapenem resistance in gram-positive cocci is commonly the result of amino acid sequence changes in their PBPs or acquisition of novel carbapenem-resistant PBPs [50],[51]. Gram-negative bacteria express carbapenem resistance through the production of β-lactamases, alterations/mutations of PBPs, porin loss as well as efflux pumps [47],[48]. A decreased expression of PBPs in *A. baumannii* and *P. aeruginosa* may result in resistance to carbapenems [52],[53].

Bacterial outer membrane proteins (OMPs) can be classified into four families: efflux porins, gated porins, non-specific porins and substrate-specific porins [54]. The function of bacterial porins is to allow the movement of molecules into and out of the cell [54]. The main families responsible for mediating carbapenem resistance are efflux porins, non-specific porins and substrate-specific porins [48],[55]. The loss or decreased expression of non-efflux porins reduces carbapenem entry into the bacterial periplasm, a phenomenon seen in *E. coli, K. pneumoniae, E. aerogenes, S. marcescens, C. freundii, S. dysenteriae* and *S. enterica* [48],[55]. Carbapenem resistance mediated by the overexpression of efflux porins is commonly reported in *E. aerogenes, A. baumannii* and *P. aeruginosa* [48]. Efflux porins in bacteria act as pumps that expel antimicrobials from the bacteria, allowing the bacteria to resist antimicrobial treatment. Carbapenem resistance mediated through the overexpression of efflux pumps is due to mutations of transcriptional regulatory proteins [56].

Carbapenems are stable against most β-lactamases, including ESBLs and AmpC β-lactamases, allowing carbapenems to have a greater bactericidal effect against gram-negative bacteria that are usually resistant to other β-lactams [57]. Beta-lactamases are periplasmic enzymes that hydrolyse β-lactam antibiotics (like carbapenems), inhibiting the antimicrobial agent from reaching the PBP target [48]. Carbapenemases are certain β-lactamases that can hydrolyse carbapenem antibiotics. The production of carbapenemases seems to be the primary cause of widespread carbapenem resistance, since the first documentation in literature occurring in various bacterial species [48],[58],[59]. Pathogens of the order *Enterobacterales* were once simple to treat, but the family of gram-negative bacteria are rapidly obtaining ESBLs that are fast becoming resistant to third- and fourth-generation cephalosporins [57],[60]. *Enterobacterales* can express several β-lactamases, becoming clinically resistant to antibiotic treatments [61]. Specific β-lactamases, which can be present in these pathogens can result in superior hydrolysis of carbapenems [32]. Such β-lactamases include Class A enzymes, Class B MBLs and Class D enzymes [32].

## 4. Carbapenemases

**Molecular and functional classification:** Beta-lactamases are classified by two differentiating properties, molecular and functional [17],[62]. Functional classification was previously done by isolating and biochemically analysing β-lactamase proteins to determine their isoelectric points, inhibition traits and substrate hydrolysis [43]. Over many years the functional classification process allowed for β-lactamases to be categorised into four functional groups, groups 1 to 4, with many subgroups within group two which are differentiated based on inhibitor characteristics or group-specific substrates [63]. Carbapenemases are functionally classified into β-lactamase groups 2f and 3 [17],[32].

Based on amino acid homology, β-lactamases are classified into four molecular classes, namely class A, B, C and D [32],[58]. Molecular classes A, C and D consist of β-lactamases that contain serine at their active sites, while class B contains metalloenzymes with zinc at their active sites [17],[32]. Carbapenemases are categorised into molecular classes A and D (serine-dependent carbapenemases) and class B (zinc-dependent carbapenemases).

**Molecular class A:** Molecular Class A carbapenemases of functional group 2f were first identified over 20 years ago and have since then been known to sporadically appear in clinical isolates in *S. marcescens, Klebsiella* species and *E. cloacae* [17]. Serine carbapenemases of class A consist of chromosomally encoded enzymes such as NMC (non-metalloenzyme carbapenemase), IMI (imipenem-hydrolysing β-lactamase) and SME (*Serratia marcescens* enzyme) as well as plasmid-encoded enzymes such as GES (Guiana extended spectrum), IBC (integron-borne cephalosporinase) and KPC (*Klebsiella pneumoniae* carbapenemase) [17]. The most clinically relevant genes of this molecular class include the KPC and GES types [28],[62],[64].

**Molecular class B:** The molecular class B, *i.e*., MBLs, is distinguished by its capability to hydrolyse carbapenems and by its susceptibility to inhibitors. Class B carbapenemase members do not utilise a covalent catalytic mechanism, allowing them to resist most commercial β-lactamase inhibitors although they are susceptible to metal ion chelator inhibitors [65]. The first reports of MBLs documented chromosomally encoded enzymes occurring in opportunistic and environmental pathogens such as *Stenotrophomonas maltophilia*, *Aeromonas* species and *Bacillus cereus* [17]. Infections related to these MBL-producing organisms, with the exception of *S. maltophilia*, are uncommon as they are commonly opportunistic bacteria and because the chromosomal genes encoding for MBLs are not readily transferable [17]. Class B of β-lactamases consists of SPM (Sao Paulo metallo-β-lactamase), GIM (German imipenemase), SIM (Seoul imipenemase), IMP (Active on imipenem), VIM (Verona integron-encoded metallo-β-lactamase) and NDM (New Delhi metallo-β-lactamase) enzymes positioned within integron structures. These enzyme integrons can occur in transposons or plasmids, increasing the likelihood of genetic information transfer between bacteria [17],[65]. The most clinically relevant β-lactamases in this class are the VIM, IMP and NDM enzymes.

**Molecular class D:** Plasmid-encoded OXA (Oxacillin-hydrolysing) serine-carbapenemases are classified under molecular class D of β-lactamases. OXA carbapenemases can functionally be characterised as penicillinases with the ability to hydrolyse cloxacillin and oxacillin, and commonly occur in *P. aeruginosa* and *Enterobacterales* [17],[63].

**Dissemination ability:** Chromosomal carbapenemases in bacteria may have initially evolved as a defensive mechanism to protect their cell wall against external threats, however, β-lactamases may also contribute to regulating cell wall synthesis [65]. The global spread of carbapenemase-producing bacteria is promoted by the association of these enzymes with acquired genetic determinants [11]. Genes encoding for carbapenemase production are more easily transferable when the gene is positioned within mobile elements such as integrons and plasmids [17]. Plasmids are categorised based on their incompatibility (Inc) or replicon groups, which stem from the replication factors exhibited by the plasmid within bacteria [66]. Each Inc group is associated with different carbapenemases, which is likely to change over time [66]. There are 28 types of plasmids that occur in *Enterobacterales*, identified by methods such as PCR-based replicon typing. Frequently occurring types such as IncA/C, IncF, IncH, IncI, IncL and IncN plasmids, express the greatest variety of resistant genes [67]. IncA/C, IncX, and IncF show global prevalence — with the latter (a narrow host range plasmid) being the most prevalent. IncF plasmids are also common in *E. coli* [68]. Additional types reported worldwide include IncP, R, U, X and W [66].

KPCs are often found on plasmids associated with the IncF [66],[69], IncN [69] and IncA/C [70] replicon types, which are known for carrying multiple resistance genes. NDM genes have been identified on various plasmid types, including, but not limited to IncA/C [71], IncF [66], IncH [72], IncL [72] and IncN [68]. Plasmids carrying OXA-48-like genes are often associated with the IncL [66],[73] and IncX replicon types [73]. VIM and IMP genes have been identified on various plasmid types, *e.g*., IncA/C, IncN, IncH [74]. The diversity of plasmids carrying these genes highlights the ability of these carbapenemases to spread among different bacterial species.

Two of the most common producers of carbapenemase enzymes in healthcare settings are *E. coli* and *Klebsiella* species. *E. coli* sequence type (ST)-131 and *K. pneumoniae* ST258 are examples of globally distributed, multi-drug resistant high-risk clones [75]. The plasmids carried by these clones appear to create a robust setting for the persistence of genes responsible for antimicrobial resistance [68]. There are many major plasmids and clones facilitating carbapenem resistance in *Enterobacterales* [66].

Many *K. pneumoniae* STs contain KPC β-lactamase, although pandemic clones most recognised include ST258, ST11 and ST101 [66]. Other well-characterised *K. pneumoniae* high-risk clones include ST14, ST15, ST17, ST45, ST147, ST307 and ST512 [76]. In an environment where antibiotics are present, high-risk clones proliferate over susceptible clones and spread antimicrobial resistance determinants through methods like vertical and horizontal gene transfer [75]. In a review by [75], carbapenemase producing *K. pneumoniae* ST307 was said to be increasing in certain regions like Italy and Columbia where it was replacing the high-risk ST258 clone. This study also reported on a 61% prevalence of ST307 in a South African clinical study (from 2014–2016).

Epidemic clones such as *K. pneumoniae* ST11 and ST147, and *E. coli* ST131 and ST101, are recognized for containing NDM genes, in addition to other β-lactamase and antibiotic resistance genes [77]. Although NDM and OXA-48 β-lactamases are more common to *E. coli* ST131, less common VIM, KPC and IMP β-lactamases were also reported in this species in some studies [68]. *E. coli* ST648, a high-risk pandemic clone, was reported in studies, each containing *bla*_NDM–5_ [78],[79]. Other researchers looking at prevalence of STs among clinical *E. coli* reported ST131, ST405, ST648, ST410 and ST167, with ST167 most significantly associated with carbapenem resistance [80].

Adding to the increasing prevalence of carbapenemase worldwide, novel carbapenemases initially identified predominantly in clinical settings are also frequently being observed in environmental settings [48],[81]. For instance, the identification of *P. pseudoalcaligenes* producing VIM-2 enzymes from a wastewater system of a hospital [81]. A study detected bacteriophages containing genes encoding for OXA-type carbapenemases from sewage, which is an example of a vector for gene transfer between organisms [82]. The dissemination of β-lactamase genes may occur in two directions, with environmental sources providing genetic material for the development of these β-lactamases and the dispersal of this genetic material in clinical strains from hospital settings into surrounding environments [17]. Furthermore, it was noted that mutual antimicrobial-resistant genes and antimicrobial-resistant gene determinants were found across three settings (environmental, clinical and farm settings) [30]. The One Health concept aims to ensure and improve public health by focusing on three primary domains: environmental, agricultural and clinical settings; thus, it is essential to understand the flow of resistance between all three domains [83]. Carbapenem resistance in bacteria is spread across all origins as further highlighted in this review.

## 5. Global dissemination of carbapenemase-producing *Enterobacterales* (CPE)

The worldwide dissemination of CPE is rapidly increasing, particularly because CPE can acquire resistance through the horizontal transfer of genes carried by mobile genetic elements [58],[84]. Horizontal gene transfer, also known as lateral gene transfer, is the passing of one or more genes via pathways other than parent-to-offspring. Horizontal gene transfer commonly occurs in bacteria via bacterial conjugation, allowing bacteria of different species to share genes. Infections by *Enterobacterales* that produce carbapenemases are linked to mortality rates as high as 67% depending on the enzyme type [85]. Most genes expressing carbapenemase production are plasmid-mediated, with frequently occurring genes such as VIM, NDM, OXA, IMP, GES and KPC types [45],[84]. Disturbingly, these enzyme types can hydrolyse almost all β-lactam agents other than carbapenems, increasing the selection pressure as well as the probability of persistence [84]. Carbapenemase production commonly occurs in *A. baumannii*, *P. aeruginosa* and species of the order *Enterobacterales*. Carbapenemase enzymes in *Enterobacterales* strains were first identified in Europe in the 1990s [86]. Nowadays, resistance to carbapenems via the production of carbapenemases in *Enterobacterales* is of great interest due to their sudden global emergence and unique dissemination [58],[84].

Initially, ESBL-producing organisms appeared relatively simultaneously in South America (Argentina) and Europe (Germany), while carbapenemase producers were mainly prevalent in North America (USA) and Asia (India and Japan) [87]. Soon ESBLs and carbapenemase producers spread globally, with outbursts occurring in many countries, including some African countries [88]. In the year 1996, *Enterobacterales* with KPC enzymes were first revealed in the USA (North Carolina) and rapidly found their way to Greece in Europe [87]. VIM-1 enzymes were discovered in *E. coli* isolates from Greece and later spread to other European countries via *K. pneumoniae* isolates, resulting in endemics [87]. *K. pneumoniae* with OXA-48 enzymes emerged in Turkey and eventually spread to other Mediterranean countries [88]. Carbapenemases are frequently found in *K. pneumoniae* followed by *E. coli* and then other genera, with a higher prevalence in Asia and southern Europe when compared to the rest of the world [88]. It has been suggested that the future dissemination of CPE will dominate hospital settings globally through *K. pneumoniae* expressing several carbapenemases, including OXA-48, NDM, KPC and VIM types [87]. The chief spread of CPE within community settings was suggested to occur via *E. coli* expressing both OXA and NDM types [87].

CPE prevalence varies across Europe, with higher prevalence found in Israel, Italy, Turkey and Greece and lower prevalence in Switzerland, the Czech Republic, Germany and Nordic countries [87]. The relations between countries can be associated with the dissemination of CPE across Europe, the cross-border movement of tourists, refugees and patients playing a vital role. An example of such dissemination is when OXA-48 spread to Belgium and France from North Africa or the identification of the Indian-originated NDM-1 in the UK [87]. The CPE epidemic is expected to increase significantly in the future, with the frequency of carbapenemase reservoirs rising worldwide. CPE primarily occurs in hospital and public health settings, although CPE dissemination in communities as well as agricultural settings is also increasing. Community-acquired NDM-1 infections have not only been associated with clinical settings but also with public water supplies [89]. In January 2008, the first case of NDM was reported in a clinical *K. pneumoniae* isolate from a Swedish patient in New Delhi (India) [90]. Following the detection of NDM-producing *K. pneumoniae*, the Swedish patient was shortly colonised with an *E. coli* isolate also capable of producing the NDM carbapenemase [90]. NDM carbapenemases have since then been found worldwide with common detection in *E. coli* and *K. pneumoniae* [90]. An occurrence of NDM-producing *E. coli* was also reported between New Zealand and Australia [91]. Some NDM carbapenemase variants have also been reported in other bacterial species, such as *Acinetobacter* and *Pseudomonas* species [91]. A very descriptive global map emphasizing the extensive global spread of carbapenemase genes within *Enterobacterales*, delineated by country and geographic region, is provided by [77]. On this map, the occurrence of NDM and KPC carbapenemases is marked within South Africa. While the dissemination of CPE in Europe, North America and Asia has been well documented, information regarding the extent of CPE in Africa has not yet been fully explored. Owing to the lack of antimicrobial resistance surveillance systems in Africa, comprehending the true extent of CPE is unlikely. However, there are already some reports of CPE occurring throughout Africa in *K. pneumoniae, E. coli, S. marcescens, Enterobacter* and *Citrobacter* species [18],[28],[29],[92]–[94]. Authors systematically reviewed articles related to CPE occurrence in Africa [18]. The most-reported carbapenemase in *Enterobacterales*, especially in North Africa, was the D class carbapenemases (primarily OXA-48). The OXA-48 enzyme was reported in various *Enterobacterales* such as *E. cloacae, E. coli, P. stuartii, K. pneumoniae* and *C. freundii* [18]. Similar findings were observed in another study, where varying OXA types occurred in four *E. cloacae* isolates in patients from Morocco, France and Turkey [95]. *K. pneumoniae* isolates producing OXA-48 enzymes reported in North African countries were also reported in France, Netherlands and Turkey [18]. OXA-23 was first reported in the UK in 1985 from a patient suffering from *A. baumannii* infection [96]. In Africa, reports of OXA-23-producing *A. baumannii* were observed in Tunisia and Madagascar [93]. A systematic review identified the presence of MBLs in all regions of Africa, as well as the presence of NDM-type enzymes, which can hydrolyse most β-lactam antimicrobial agents [18]. In this review, NDM enzymes occurring in *Enterobacterales* were reported in North African countries, including Egypt, Algeria and Libya. In another study, where NDM prevalence was observed in Africa, Algeria was described as having high rates of NDM-producing *E. coli*, while lower rates were observed in Cameroon, Egypt and SA [91].

**Figure 1.**
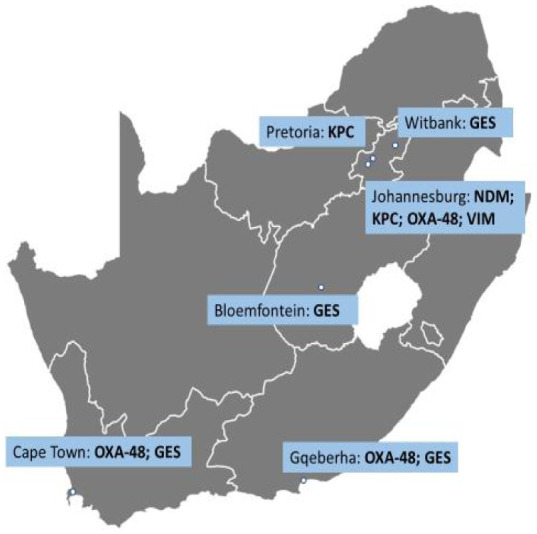
A map showing the emergence and dissemination of carbapenemase genes among *Enterobacterales* in SA. The information in this diagram was derived from [75].

The prevalence of carbapenem resistance by *Enterobacterales* and non-fermenting bacterial strains is increasing worldwide and in SA (Figure 1). This figure expands on the carbapenemases within South Africa marked on the global map by [77]. In Gauteng in 2011, the first report of NDM produced in *E. cloacae* was documented with the second case of this organism being isolated in KwaZulu-Natal less than a year later [92],[97]. Other cases of CPE include the detection of OXA-48 and OXA-181-producing *K. pneumoniae* in Gauteng in 2011 [97]. CPE outbreaks have also occurred in SA, such as the 2012 outbreak in the Western Cape caused by OXA-181-producing *K. pneumoniae* and an Eastern Cape outbreak caused by VIM, IMP and OXA-48-producing *E. cloacae* [97].

A study examined the presence of CPE in SA between the years 2012 and 2015 [97]. The authors investigated 1193 isolates suspected of carbapenemase presence from laboratories across SA. At least one or a combination of genes coding for carbapenemases were found in 812 (68%) isolates. From these 812 isolates, *bla*_NDM_ genes (57%) predominated followed by *bla*_OXA_ (26%), *bla*_VIM_ (10%), *bla*_IMP_ (4%), *bla*_GES_ (2%) and *bla*_KPC_ (1%); the highest occurrence of carbapenemase genes was detected in *Klebsiella* species (71%) followed by *Enterobacter* species, *S. marcescens, E. coli, Citrobacter* species, *Providencia* species, *Morganella morganii* and *Raoultella* species. The presence of carbapenemase genes in *S. marcescens, Providencia* species and *M. morganii* is distressing as these organisms are intrinsically resistant to colistin, which is an antimicrobial agent used to treat carbapenem-resistant infections, therefore making treatment of such infections even more challenging. This study concluded an observed 40% increase in CPE occurring in SA over the three-year period of the study.

In another study where carbapenem resistance in SA was analysed, the author identified approximately 2315 cases that occurred between January 2000 and May 2016 [22]. The author observed that these cases predominantly occurred in the Gauteng province, followed by the KwaZulu-Natal province. Carbapenem resistance frequently occurred in *K. pneumoniae* followed by *A. baumannii, E. cloacae* and *S. marcescens*. The author noted that the most commonly occurring carbapenemase gene was NDM followed by OXA-48 [22],[28].

Researchers molecularly characterised 45 carbapenem-resistant isolates from 10 varying private hospitals in SA between 2012 and 2013 and found that 41 of the isolates carried genes encoding for carbapenemase production. Most of the isolates were *K. pneumoniae* followed by *S. marcescens, Enterobacter* species, *K. michiganensis* and *C. freundii* [28]. Whole genome sequencing revealed carbapenemase genes with NDM-1 occurring the most followed by GES-5, NDM-5 and OXA-232 [28]. Another study identified carbapenemase-producing *Acinetobacter* species in an academic hospital in KwaZulu-Natal [98]. The findings from this study suggested that OXA-23 genes spread between two patients, between the intensive care unit and the vascular unit.

Several factors are associated with an increased risk of CPE dissemination such as global travel, unhygienic practices in a community, or healthcare exposure and the overuse and misuse of antibiotics being the main contributing factor. Such injudicious use of antimicrobial agents leads to increased pressure for selecting resistant bacteria, including CPE. The spread of CPE in Africa may be occurring for several reasons, for instance, the over-the-counter overuse of antimicrobials with many countries showing little regard to antibiotic stewardship. Egypt, along with Pakistan and India, has reported an increased sale of over-the-counter carbapenems according to reports from The Lancet Infectious Diseases Commission [99]. This may be the result of relaxed governing of antimicrobial use, the lack of good antibiotic stewardship systems and the availability of affordable generic carbapenem antibiotics [18],[99]. CPE can also occur in agricultural settings, with research reporting many cases of CPE presence in food and companion animals from across the globe [18],[21]–[23]. Currently, there are very few studies regarding CPE dissemination in agricultural settings in Africa [24],[27],[29],[100]–[102], indicating the need for CPE surveillance in livestock settings as well as the food chain.

## 6. Food production and livestock

Production of livestock is one of the fastest-growing sectors in the agricultural industry, with over 70% of agricultural land being used for livestock production in SA [103]. Bacterial ABR is ubiquitous in varying environments from aquaculture sectors, food processing settings and food-producing animals to wildlife and companion animals [104]. High volumes of antibiotics are used in meat-producing industries compared to the volumes used in human medicine [31],[100]. These industries depend on antibiotics to improve productivity and animal health, particularly in intensively reared animals [100]. The highest volumes of antibiotics are used for intensively reared (a method that uses higher inputs and more advanced agricultural techniques) pigs and poultry, while extensively farmed (a method that requires much more land for production) animals such as cattle, goats and sheep use lower volumes of feed as they mainly feed on grass, have lower herd densities and are seen as healthier animals [105]. The inappropriate use of antibiotics in meat-producing industries has led to great selective pressure, increasing the possibility for bacteria to develop resistance and produce larger resistant populations [16],[31],[106].

Food products of animal origin have been identified as potential sources for antibiotic-resistant organisms, however, there is limited research on the presence of CPE in food and agricultural settings. Carbapenem drugs are prohibited in Europe; although special exceptions may be allowed for companion animals [107]. A recent review on CPE in food-producing, companion and wildlife animals between the years 1980 and 2017 has identified an increase in CPE within the food and agricultural sectors. This study identified 68 articles describing the CPE in the three categories of animals as well as five predominantly occurring genes that express carbapenemase production; VIM, KPC, NDM, OXA and IMP genes [23]. Furthermore, two articles described that 33 to 67% of exposed farm workers on poultry farms carried CPE associated with isolates from the farm environment [23].

**Figure 2.**
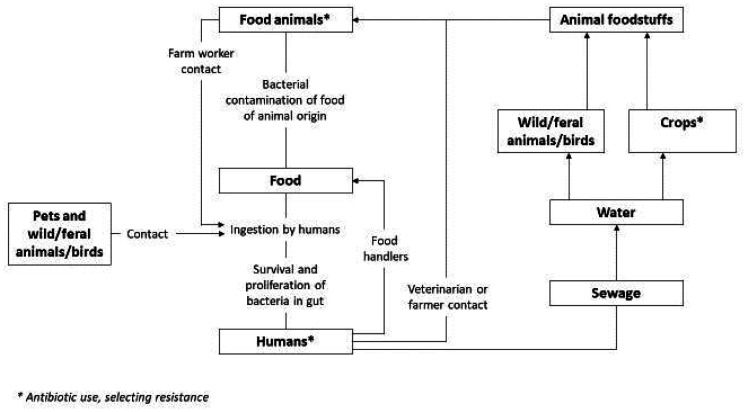
Possible transmission routes of antibiotic-resistant pathogens and determinants between humans and animals. The information in this diagram was derived from [109].

Alarmingly, many antibiotics considered vital in human medicine such as sulphonamides, penicillins and tetracyclines are also being used by food animal-producing industries [104]. Various reports have indicated that non-therapeutic antimicrobials used in food-producing animals have been linked to bacterial resistance in people who live close to farms as well as the general population through the food chain [23],[108]. Identifying the link between antibiotic-resistant bacteria in humans and animals is difficult due to the many possible routes of transmission, including through animals used in food production [109]. An example of a direct route of transmission would be human contact with wildlife and companion animals, and indirect routes include contamination of streams, rivers and soils by farm effluents (Figure 2).

These indirect routes could result in cross-contamination of water and crops used for food preparation and drinking [104]. However, the main route of transmission of bacterial ABR has been hypothesised to be the food chain. Animals used in food production have been identified as reservoirs for antibiotic-resistant bacteria, whereas the presence of carbapenem-resistant bacteria in livestock has only recently been studied [110]. Reports of CPE occurring in livestock and seafood in European, American, Asian and African countries were reported in a systematic review [23]. One study identified OXA genes encoding for carbapenemase production from livestock in the genus *Shewanella*, which were not affiliated with mobile genetic elements, indicating a low risk for foodborne outbreaks [23]. However, cases relating to plasmid-enclosed carbapenemase genes such as OXA in *E. coli* from pigs in Italy and VIM in *E. coli* from broilers in Germany are more of a concern as these enzymes are also predominantly found in CPE human infections in Germany and other European countries [111]. Similar findings of CPE dissemination in livestock have been seen in countries across the globe such as India, China and Algeria [19],[23],[24],[112],[113].

A recent study reported the presence of ESBL-producing organisms from cattle-related samples in the Northwest province of SA [101]. The authors isolated and characterised ESBL-producing *K. pneumoniae* and *E. coli* isolates from 151 cattle-related samples and identified that 53.1% of the isolates contained genes expressing for ESBLs [101]. These findings in agricultural settings are not unexpected as numerous CPE isolates have been detected in environmental and clinical settings in SA, increasing the likelihood of CPE presence in agricultural settings [22],[24],[27],[29],[97],[98],[101],[114].

**Porcine:** The earliest detection of CPE in food-producing animals dates to 2011 on a pig farm in Germany, where an *E. coli* strain with the VIM-1 gene was isolated from a pig [111]. The isolate was resistant towards cephalosporins, cephamycin, amoxicillin/clavulanic acid and penicillin — however, this isolate showed intermediary susceptibility to carbapenems (meropenem, ertapenem and imipenem). Polymerase chain reaction (PCR) results later revealed that the isolate carried both *bla*_ACC-1_ (AmpC-encoding gene) and *bla*_VIM-1_ genes within its integron [111]. Although this German VIM-producing *E. coli* was the first CPE detected in pigs, its sequence type (ST88) had been previously detected in cattle, chickens and humans in Germany. Furthermore, this VIM-producing *E. coli* strain was found broadly disseminated on the pig-fattening farm tested in 2011 — showing that the persistent spread of CPE in farm settings is possible [115]. Another German study between the years 2011 to 2012 revealed the presence of VIM-producing *S. enterica* infantis serovars from one broiler farm and two pig farms [21]. The isolated pathogens were also identified to express both *bla*_ACC-1_ and *bla*_VIM-1_ genes. *Salmonella* infantis is one of the top five *Salmonella* serovars that are responsible for salmonellosis in humans in Europe, characterised by septicaemia and even death [116]. Consequentially, the findings on carbapenemase-producing *Salmonella* infantis in poultry and pigs are quite concerning as farm settings can be considered as *Salmonella* infantis reservoirs [116]. Carbapenems are not authorised for use in German animal husbandry [117], suggesting that the source of the *bla*_VIM-1_ gene may be due to selective pressure caused by the authorised use of third-generation cephalosporins [19]. The presence of genes expressing resistance to commonly used antimicrobials in pig production may be favoured by the low selective MIC (minimum inhibitory concentrations required to inhibit the growth of 90% of organisms) values—therefore, even minimal antimicrobial concentrations used in livestock production and human treatment can likely result in sufficient selective pressure to select and maintain plasmids encoding for resistance in microbial populations [118]. A recent occurrence of the *bla*_OXA-181_ gene in *E. coli* isolated from two pigs on an Italian farm was described [119]. Each pig contained two unrelated strains, belonging to sequence types ST641 and ST359. The *bla*_OXA-181_ gene was located on a plasmid that showed high similarity to human and other animal plasmids, indicating that ABR plasmids are widely disseminated in *E. coli* strains [119]. Furthermore, the incidence of plasmid-borne carbapenemase genes in *Enterobacterales* was also found in the USA. From faecal and environmental samples obtained on pig farrowing and nursery barns, isolates harbouring the *bla*_IMP-27_ gene were found among organisms from *Enterobacterales* [25]. The use of cephalosporins on the farm was observed. Further, a higher prevalence of isolates expressing the IMP gene was found in the farrowing barn compared to the nursery barn [25]. The dissemination of CPE in food products and food animals is a global phenomenon even reaching the Far East, with a *bla*_NDM-1_ gene being isolated from *A. baumannii* in south China [120]. The isolate was detected in the lungs of a pig suffering from sepsis and pneumonia, reared on a farm whereby varying cephalosporins, quinolones, aminoglycosides and β-lactams were frequently administrated. Carbapenem use in food-producing animals in China is unauthorised, however, other β-lactam agents are permitted for use [121]. The use of other β-lactams (non-carbapenems) may facilitate the presence of carbapenemase-producing genes in food animals [122]. Lastly, and quite worryingly, is the detection of Shiga toxin-producing *E. coli* expressing the *bla*_NDM-1_ gene in piglets in India. The Indian farm had a history of using several antimicrobial drugs, as well as third-generation cephalosporins [123].

**Bovine:** The first reports of carbapenemase genes detected in cattle were from dairy cattle isolates of the *Acinetobacter* genus carrying the *bla*_OXA-23_ gene in France in 2010 [124]. The *bla*_OXA-23_ gene is widely found in *A. baumannii*, which is an opportunistic nosocomial pathogen that has become one of the most important multi-drug-resistant bacteria occurring in hospitals globally [125]. Strains of *Acinetobacter* species in Germany have been reported to carry chromosomal *bla*_OXA-23_ genes that were isolated from cattle nasal swabs [126]. In another case in the USA where third-generation cephalosporins were frequently used on a dairy cattle farm, an *A. baumannii* isolate was discovered that harboured the novel *bla*_OXA-497_ gene, which is included in the OXA-51-like group of enzymes [127]. In China, the first report of *K. pneumoniae* carrying *bla*_NDM-5_ genes in cattle was detected in faecal and milk samples of dairy cattle suffering from mastitis [19]. However, *E. coli* and *K. pneumoniae* harbouring *bla*_NDM-5_ genes were previously detected in human patients from several countries. Again, the impact of a variety of β-lactam antimicrobials in the Chinese dairy industry may have contributed to selective pressure for the occurrence of *bla*_NDM-5_ genes in *K. pneumoniae* isolates [19]. A South African study assessed 233 cattle faecal samples for the presence of CRE, whereby 151 CRE isolates were genotypically screened for the presence of six carbapenemase genes: KPC, OXA-23, OXA-48, NDM, VIM and GES genes [24]. From the 151 CRE isolates assessed, 94.7% were found to harbour one or a combination of the genes. The most frequent encountered were KPC (35.8%), NDM (20.5%) and GES (17.9%) genes [24].

**Poultry:** The first reports of presumptive carbapenemase-producing *E. coli* in broilers were from Cyprus and Romania in 2016 [128]. The Romanian *E. coli* isolates were observed to be harbouring *bla*_OXA-48_ genes [128]. The majority of the studies regarding CPE in poultry have been reported in Africa and China [19]. In China, *A. lwoffii* producing the NDM-1 carbapenemase from chicken anal swabs, expressed resistance towards eight of nine β-lactam antimicrobials, including ertapenem, meropenem and imipenem. Also, from China, *S. indiana* isolates harbouring *bla*_OXA-1_ and *bla*_NDM-1_ genes were reported from a chicken carcass at the slaughtering stage of processing. The strain carried other β-lactamases and expressed resistance to multiple drugs including phenicol, aminoglycosides and more [121]. The occurrence of highly resistant *Salmonella* species is of great concern for human health, owing to the genus's zoonotic attitude and wide dissemination in the food chain [19]. In an Egyptian study, carbapenemase-producing *K. pneumoniae* isolates were examined from broiler chickens reared on varying farms, as well as the drinking water and farm workers [129]. Carbapenemase-producing *K. pneumoniae* isolates were detected in 6% of the water samples and 15% of the broiler samples screened. All 15 poultry isolates were positive for the *bla*_NDM_ gene, with 11 of the isolates carrying *bla*_NDM_, *bla*_OXA-48_ and *bla*_KPC_ genes and 4 of the isolates carrying either *bla*_OXA-48_ and *bla*_NDM_ genes or *bla*_KPC_ and *bla*_NDM_ genes [129].

**Seafood:** ABR surveillance programs in Western countries primarily screen poultry, pork and beef for antibiotic-resistant organisms and focus on *Salmonella, E. coli, Enterococcus* species and *Campylobacter* species [110]. This method of surveillance does not represent the diverse range of food animal products such as seafood. To address this gap, researchers screened 121 seafood products in retail stores in Canada that originated from Asia [110]. The authors found 4 seafood samples (sea squirt, squid, seafood medley and clams) that carried carbapenemase-producing bacteria (all *bla*_OXA-48_) [110]. The organisms that were found to have the carbapenemase gene included *Stenotrophomonas, Pseudomonas* and *Myroides* species [110]. Antimicrobials are also often used in intensive aquaculture farming, which is a phenomenon that supports the occurrence of bacterial ABR in seafood products. In addition, fish can acquire ABR bacteria through polluted seawater contaminated by agricultural drains or sewage [19]. In the Mediterranean Sea in 2013, *A. baumannii* isolates carrying *bla*_OXA-23_ and *bla*_OXA-51_ genes were identified from two fish (*Pagellus acarne*). The isolate was resistant towards third-generation cephalosporins, aminoglycosides and carbapenems [130]. A recent detection of a VIM-producing *E. coli* (ST10) was isolated in Venus clams in Italy, which is worrying as these clams are commonly eaten raw, allowing for easier transmission of CPE from foodstuffs to the consumers [113].

The transmission of resistant bacteria through the food chain to humans is of great concern, highlighting the importance of good food handling practices and safe preparation by producers and consumers to prevent antibiotic-resistant bacterial infections [131]. One of the processing steps where resistant bacteria can be introduced into the food chain is the slaughtering step. Spraying butchered carcasses with organic acids can be used as a technique to remove unwanted pathogens, but it may also result in the development of acid-resistant bacteria [104]. Another mechanism for bacterial infection in humans is the consumption of residual antibiotics in meat products leading to the development of bacterial ABR within the human gut [132]. There are various techniques which can be put into place to decrease the development of bacterial ABR in the food chain. Decreasing the non-therapeutic metaphylaxis in meat-producing industries, increasing good farm practices and hygiene and education regarding the judicious use of antibiotics are a few examples of steps that could aid in slowing down the development of bacterial ABR [105],[133].

## 7. Concluding remarks

The past few decades have seen the rapid emergence and spread of antimicrobial resistance, including the resistance to carbapenem antibiotics, which are the last line of defence drugs against multi-drug resistant bacteria.

CPE can also occur in agricultural settings, with research reporting many cases of CPE presence in food- and companion animals across the globe. Compared to clinical data, there are very few studies regarding CPE dissemination in livestock settings as well as the food chain in Africa. More research is required to reveal the extent of CPE dissemination in SA.

## Use of AI tools declaration

The authors declare they have not used Artificial Intelligence (AI) tools in the creation of this article.
